# The discovery of dynamic chiral anomaly in a Weyl semimetal NbAs

**DOI:** 10.1038/s41467-020-14749-4

**Published:** 2020-03-06

**Authors:** Xiang Yuan, Cheng Zhang, Yi Zhang, Zhongbo Yan, Tairu Lyu, Mengyao Zhang, Zhilin Li, Chaoyu Song, Minhao Zhao, Pengliang Leng, Mykhaylo Ozerov, Xiaolong Chen, Nanlin Wang, Yi Shi, Hugen Yan, Faxian Xiu

**Affiliations:** 1 State Key Laboratory of Surface Physics and Department of Physics, Fudan University, Shanghai, 200433 China; 2 State Key Laboratory of Precision Spectroscopy, East China Normal University, Shanghai, 200062 China; 3 Institute for Nanoelectronic Devices and Quantum Computing, Fudan University, Shanghai, 200433 China; 4 Department of Physics, Cornell University, Ithaca, 14853 NY USA; 5 International Center for Quantum Materials, School of Physics, Peking University, Beijing, 100871 China; 6 School of Physics, Sun Yat-sen University, Guangzhou, 510275 China; 7 Department of Physics, University of California at Berkeley, Berkeley, 94720 CA USA; 8 Collaborative Innovation Center of Quantum Matter, Beijing, 100871 China; 9 Beijing National Laboratory for Condensed Matter Physics, Institute of Physics, Chinese Academy of Sciences, Beijing, 100190 China; 10 State Key Laboratory for Artificial Microstructure and Mesoscopic Physics, Beijing Key Laboratory of Quantum Devices, Peking University, 100871 Beijing, China; 11 National High Magnetic Field Laboratory, Florida State University, Tallahassee, FL 32310 USA; 12 National Laboratory of Solid State Microstructures, School of Physics, Nanjing University, Nanjing, China; 13 Collaborative Innovation Center of Advanced Microstructures, Nanjing, 210093 China; 14 Key Laboratory of Micro and Nano Photonic Structures (Ministry of Education), Fudan University, Shanghai, 200433 China

**Keywords:** Topological insulators, Electronic properties and materials

## Abstract

The experimental discovery of Weyl semimetals offers unprecedented opportunities to study Weyl physics in condensed matters. Unique electromagnetic response of Weyl semimetals such as chiral magnetic effect has been observed and presented by the axial *θ* **E** · **B** term in electromagnetic Lagrangian (**E** and **B** are the electric and magnetic field, respectively). But till now, the experimental progress in this direction in Weyl semimetals is restricted to the DC regime. Here we report experimental access to the dynamic regime in Weyl semimetal NbAs by combining the internal deformation potential of coupled phonons with applied static magnetic field. While the dynamic **E** · **B** field is realized, it produces an anomalous phonon activity with a characteristic angle-dependence. Our results provide an effective approach to achieve the dynamic regime beyond the widely-investigated DC limit which enables the coupling between the Weyl fermions and the electromagnetic wave for further study of novel light-matter interactions in Weyl semimetals.

## Abstract (web summary)

Unique electronmagnetic response of Weyl semimetals have only been reported in static field regime. Here, the authors report evidence of a dynamical chiral anomaly response realized by internal collective lattice deformation with an external static magnetic field in a Weyl semimetal NbAs.

## Introduction

Chiral anomaly designates the breaking of chiral symmetry upon the quantization of relativistic fermionic particles, giving rise to the non-conservation of chiral charges^1–5^. The discovery of Weyl semimetals offers a great opportunity to study this chiral anomaly in condensed matters due to the definite chirality carried by the Weyl fermions^6–12^. Featured by the band crossings in three-dimensional reciprocal space, Weyl semimetals host low-energy electronic excitations that mimic the Weyl fermions with well-defined chirality.

**Fig. 1:**
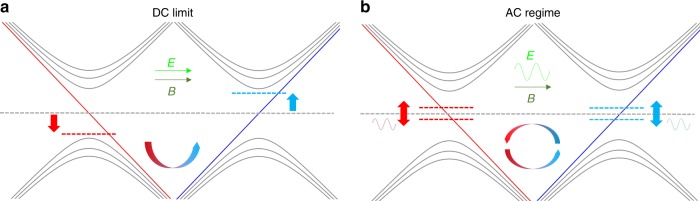
Chiral anomaly in the DC and AC limit in Weyl semimetals. **a** Landau level energy versus momenta (*k*) for chiral anomaly in the DC limit. Chiral anomaly is induced by static **E** and **B** fields, resulting in a static shift of the Fermi level and a constant chiral current. Gray solid lines are the non-chiral Landau levels. Red and blue lines are the chiral Landau levels with opposite chirality. Dashed lines and arrows (in red and blue) indicate the shifts of the Fermi level. The arrow in the middle is the chiral magnetic current. **b** Landau level energy versus *k* for chiral anomaly in the AC limit. The sine wave in green denotes the time-varying **E** field parallel to the external **B** field which induces the dynamic chiral anomaly. Both the chiral charge and chiral current are time-varying and covariant, leading to the interplay between the chiral anomaly and phonon modes.

The unique electromagnetic response of Weyl semimetals compared to other materials can be understood by *θ***E** · **B** in electromagnetic Lagrangian, where *θ* is determined by the position and chemical potential shift of Weyl nodes^11^. A non-vanishing *θ***E** · **B** field in Weyl semimetals leads to additional charge and current which corresponds to anomalous Hall effect and chiral anomaly, which have been extensively studied^11–19^. In the presence of parallel **E** and **B** fields, chiral anomaly leads to the charge pumping between Weyl nodes with opposite chirality as illustrated in Fig. 1a. Owing to the opposite moving directions and unequal occupation of chiral charges, a net current parallel to the **E** field will be induced, which can be detected as the negative longitudinal magneto-resistance in transport experiments^13,15,17–19^.

Although the *θ***E** · **B** presents a fundamental difference in electromagnetic response between Weyl semimetals and others, till now, most experimental progress related to *θ***E** · **B** such as chiral anomaly, was achieved in the DC limit where **E** · **B** is static. Experimental access to the dynamic **E** · **B** term could reveal the unique and rich character of Weyl fermions in the AC regime and allow for the coupling between the Weyl fermions and light^11^. Although desired, achieving oscillating **E** · **B** with the external field is challenging due to the semi-metallic nature of the materials (see Supplementary Note 1 for the experimental challenge).

Here we achieve the dynamic *θ***E** · **B** field by utilizing the electric field driven by the internal phonon potential along with a static magnetic field. Therefore, the chiral anomaly can be realized in the AC regime. As a result, an anomalous transition of the phonon mode is detected as a specific phonon activity observed in IR spectroscopy in (and only in) the presence of an external magnetic field. A series of controlled magneto-infrared experiments also show that such a phonon activity is dependent on the relative angle between the magnetic field and the polarization direction of the light. These observations fully agree with our theory based on the symmetry analysis and the prediction of dynamic chiral anomaly^20–22^.

## Results

### Dynamic chiral anomaly

The lattice dynamics in crystals are characterized by the mode-effective phonon charge **Q**. The phonon mode is infrared active in the optical spectra only if the electric field of the probing light **E**_L_ satisfies **E**_L_ **· Q** ≠ 0. At the same time, the phonons represent lattice vibrations and give rise to dynamic deformation potentials for the electrons via electron–phonon interaction. In Weyl semimetals, together with a static magnetic field **B** applied externally, this oscillating internal **E** field may generate an AC **E** · **B** field and result in a dynamic chiral magnetic current and charge pumping. In return, such active electronic responses modify the dynamics of the phonons^21,22^, which obtains an additional phonon effective charge δ**Q** ∝ **B**∑*λv*_F||_ after the Weyl fermions are integrated out^20^. Here the summation is over the Weyl nodes, v_F||_ represents each Weyl node’s chiral Landau level signed Fermi velocity in the presence of **B**, and *λ* is the axial electron–phonon coupling. In particular, even a phonon mode, being absent from the IR spectrum at zero field either due to **Q** ⊥ **E**_L_ or the component of **Q** parallel to **E**_L_ being too small, may become infrared-active at finite fields if the emergent δ**Q** satisfies **E**_L_ **·** δ**Q ∝ E**_L_ · **B** ≠ 0. This predicted magnetic-field-induced anomalous phonon activity and the highly anisotropic dependence between **B** and **E**_L_, are extremely rare in non-magnetic bulk materials and characteristic to the Weyl and Dirac semimetals^21,22^.

**Fig. 2:**
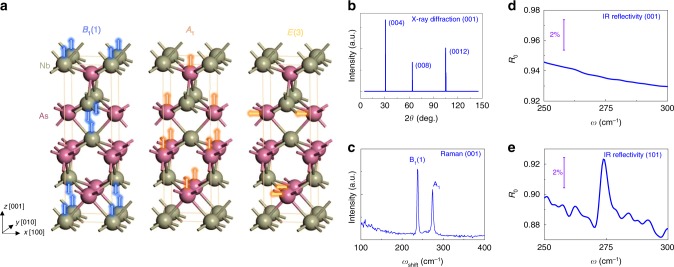
Lattice dynamics in Weyl semimetal NbAs without magnetic fields. **a** The lattice structure of NbAs. The B_1_(1), A_1_, and E(3) modes originate from the motion of different atoms. Arrows denote the displacement motion of atoms. In point group C_4v_ crystals, the A_1_ phonon and E(3) phonons are associated with the vibration along the *z*-direction and xy plane, respectively. **b** X-ray diffraction, showing the (001) surface of the crystal. **c** Raman spectroscopy, revealing the phonon mode around 275 cm^−1^. **d** Infrared reflectivity of the (001) surface. The phonon modes are absent from the zero-field spectrum. **e** Infrared reflectivity of (101) surface with well-resolved phonon resonance.

Based on these predictions, several ingredients are indispensable for realizing and probing the AC *θ***E** · **B** term and dynamic chiral anomaly, including (1) the presence of an external static magnetic field **B**, (2) a phonon probe, (3) insensitive phonon modes to **E**_L_ at zero field for contrast, and (4) a strong electron–phonon coupling. The experiment is designed accordingly in the following way. To satisfy conditions (1) and (2), we choose magneto-optical spectroscopy which has been demonstrated to be a useful tool for studying the phonon modes and topological condensed matters^23–28^. The presence of an external static magnetic field produces the chiral Landau level as one of the pre-requisites of the chiral anomaly and allows for a non-vanishing **E** · **B** term. Among all the material systems hosting the Weyl fermions, NbAs material family is selected for the experiments due to the well studied strong electron–phonon interaction^29^ and the related phonon vibrations are along the high symmetry directions so that the conditions **(3)(4)** are satisfied. The strong electron–phonon coupling, as indicated by the large signal of the A_1_ mode in NbAs, allows a large axial coupling in the presence of the magnetic-field-induced symmetry-breaking and a good resolution for the observation of the dynamic chiral anomaly effect. NbAs hosts a body-centered tetragonal structure with the broken inversion symmetry along the [001] direction as shown in Fig. 2a. Here we use X-ray diffraction (Fig. 2b) to identify the (001) surface of the as-grown NbAs crystal (see methods for growth details) where the A_1_ and B_1_ modes vibrate perpendicularly to this surface, while the E modes vibrate parallelly to the surface. The extracted lattice structure and lattice constants are in accord with Fig. 2a (see Supplementary Note 2 for details of the crystal).

### Phonon modes at zero field

Firstly, we performed Raman spectroscopy on the NbAs crystal. Figure 2c presents the Raman spectroscopy on the (001) surface with both incident and scattered light unpolarized. Prominent peaks can be witnessed around 235 cm^−1^ and 275 cm^−1^, which correspond to the B_1_(1) and A_1_ phonon modes^30,31^, respectively. The B_1_(1) mode which originates from the motion of Nb atoms is infrared-inactive regardless of the crystal surface. Figure 2d shows that the A_1_ phonon cannot be observed in the infrared reflectivity of NbAs (001) surface, while on the (101) surface, as shown in Fig. 2e, the corresponding phonon resonance can be well resolved with the same frequency as that from the Raman spectrum. It helps to confirm that the A_1_ phonon which originates from the motion of As atoms is indeed polarized along the *c*-axis (arrows in Fig. 2a), therefore, inactive in the infrared spectrum for the (001) surface and consistent with the symmetry analysis of C_4v_ crystals^30^. The E(3) phonon modes, which originate from the As atoms as well but polarized in the xy plane, are absent from the infrared spectrum for the (001) surface. It indicates that despite being allowed, the phonon charge **Q** of the E(3) phonons is inadequate for the observable response.

### Field-induced phonon activity

**Fig. 3:**
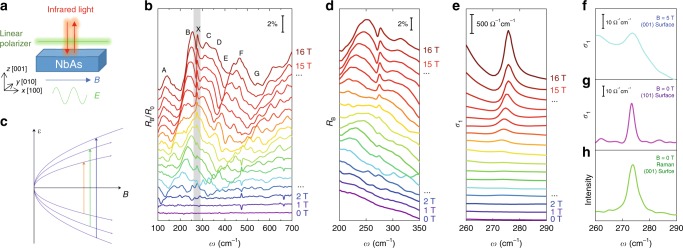
Magnetic-field-induced phonon activity. **a** A schematic plot of the experimental configuration. Reflectivity spectroscopy is performed on the (001) surface of NbAs. The polarization of the light is parallel to an external **B** field. **b** Normalized magneto-optical reflectivity spectrum *R*_*B*_*/R*_*0*_ under different magnetic fields. A–G features are typical behavior of inter-Landau-level transitions. X features come from the field-induced phonon mode. **c** A schematic plot of Landau level energy versus the magnetic field for ideal Weyl semimetal at *k*_*x*_ = 0. Arrows represent the allowed optical transitions. **d** Absolute reflectivity *R*_B_ under different **B** fields. Phonon modes become active in the presence of **B** field. **e** Real part of optical conductivity under different **B** fields. **f** Real part of optical conductivity on (001) surface with **B** = 5 T. **g** Optical conductivity on (101) surface without **B** field**. h** Raman spectrum of the (001) surface.

To study the possible phonon activity induced by the dynamic chiral anomaly, we performed magneto-infrared spectroscopy measurements in a well-designed Voigt geometry. The magnetic field is parallel to the (001) surface. The infrared light propagates perpendicularly to the (001) surface with polarizations parallel to **B** as shown in Fig. 3a. Figure 3b displays the stack view of the relative magneto-infrared spectra normalized by zero-field reflectivity (*R*_B_/*R*_0_) from 0 to 16 T. A series of peaks and dips can be observed to evolve as the magnetic field increases and can be divided into two types. First, multiple features labeled by A-G cover the entire spectrum range. Their peak frequencies increase with the magnetic field, and their widths are around 30 cm^−1^. We attribute all these A-G features to the inter-Landau-level transitions. Figure 3c depicts the Landau quantization of an ideal Weyl semimetal with a Hamiltonian *H*(*k*) = *v*_**F**_**k** · **σ** (*ħ* = 1 for notation simplicity). Here *v*_F_ is the Fermi velocity, **k** is momentum, and **σ** is Pauli matrix. Energy of the Landau levels can be expressed as $\varepsilon _n = {\mathrm{sgn}}(n)v_{\mathrm{F}}\sqrt {k_z^2 + 2\left| n \right|eB}$. In the event of photon absorption, electrons at the occupied states can be excited to the unoccupied states following selection rules denoted by the arrows for the geometry described in Fig. 3a (**E**//**B**), resulting in observable features in the optical spectrum. Owing to the larger gaps between the Landau levels at higher fields, the frequency of the absorbed photon increases with magnetic fields. The Landau level spectra of Weyl semimetal NbAs have been studied thoroughly in previous reports^32,33^. We now focus on the feature X, which shows a completely different behavior. This mode only becomes observable with magnetic fields, but unlike the inter-Landau-level transitions, its frequency does not shift with the magnetic field. The invariance of its frequency and its much narrow width indicate a different origin for the X peak. By comparing the magneto-infrared spectra with the zero-field Raman and the zero-field infrared spectrum (Fig. 2), the frequency of the X peak is found to be identical to that of the A_1_ phonon, which we argue that it is also accidentally degenerate with the E(3) phonons at **k** = 0 (see Supplementary Note 9 for details).

To directly probe the phonon behavior under magnetic fields, the absolute reflectivity spectrum of *R*_B_ (Fig. 3d) is extracted by multiplying the normalized spectrum *R*_B_/*R*_0_ (Fig. 3b) with zero field spectrum *R*_0_. From Fig. 3d, one can directly determine that the phonon mode becomes active in the presence of a magnetic field and becomes stronger at higher fields. To make the argument more rigorous, we calculate the real part of optical conductivity *σ*_1_ from the Kramers–Kronig analysis^34^ (see Supplementary Note 3 for details of extracting optical conductivity). At zero field, no phonon resonance is present (Fig. 3e). And a peak with Lorentzian-shape gradually emerges and the peak intensity monotonically increases with the **B** field, consistent with the prediction that δ**Q** increases with the **B** field^21,22^. Even at low fields, e.g., 5 T, the phonon resonance can be well resolved as shown in Fig. 3f. The tilted baseline comes from the inter-Landau level resonance, which is larger in both amplitude and width. At high fields, the line shape of the field-induced phonon mode is influenced by the nearby inter-Landau level transitions. It remains otherwise symmetric Lorentzian shape once eliminating these transitions. Detailed information of the Lorentzian line shape can be found in Supplementary Note 4 and Supplementary Figure 1. We also performed the Kramers–Kronig analysis for the zero-field spectrum at (101) surface (Fig. 3g). Without the influence from the inter-Landau-level transitions, a well-shaped Lorentzian peak from the phonon resonance can also be observed, similar to the result from the Raman spectroscopy (Fig. 3h). This phonon mode exhibits the symmetric Lorentzian line shape instead of being a Fano shape because the Fermi energy is higher than the half value of the phonon energy. Therefore, Fig. 3d–h directly show that the phonon originally being absent in the IR spectrum becomes active in the presence of a magnetic field, consistent with the prediction of phonon activity induced by dynamic chiral anomaly^21,22^.

### Angle-dependent characteristics of the field-induced phonon

**Fig. 4:**
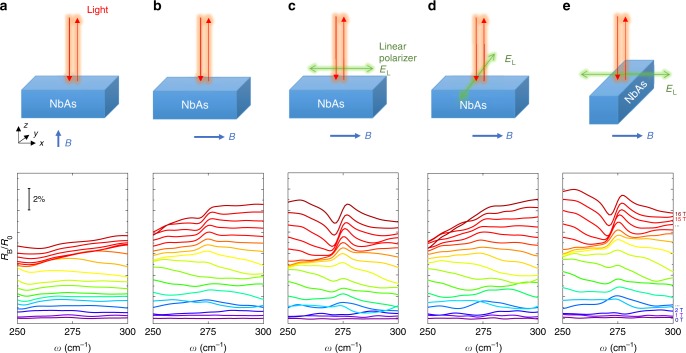
The phonon activity controlled by the direction of the magnetic field. **a**–**e** Experimental geometries (top panels) and normalized reflectivity under magnetic fields (bottom panels). Comparing **a**–**e**, we show that the phonons only activate along the **B** direction and can be observed when the oscillating **E**_L_ has a parallel component to the **B** field.

In the presence of an external magnetic field **B**, the contribution of the chiral anomaly to the electrical response is highly anisotropic due to the **E · B** term in the effective Lagrangian ${\cal{L}} = \theta {\mathbf{E}} \cdot {\mathbf{B}}$. For the dynamic chiral anomaly discussed here, the oscillating chiral magnetic current is parallel to the **B** direction. Therefore, the responsible phonon charge δ**Q** is predicted to be parallel to the direction of the magnetic field^22^. To further elucidate the direction of the phonon charge in experiments, a series of angle-dependent measurements were carried out by rotating the polarization of incident light **E**_L_ and **B** field. Here we focus on the (001) surfaces of the crystal because this is the only surface where all the phonon modes **Q · E**_L_ vanish at zero field. Figure 4a, b compares the experimental results in Faraday geometry and Voigt geometry, where the propagating direction of the light is parallel and perpendicular to **B**, respectively. The light is not polarized for both cases. In the Faraday geometry (Fig. 4a), no phonon resonance is observed. In contrast, the phonon resonance shows up around 275 cm^−1^ in the presence of a magnetic field in the Voigt geometry (Fig. 4b). An apparent difference between the two geometries is whether the **E**_L_ field is parallel to the external **B**. In the Faraday geometry, the **E**_L_ field from light is completely orthogonal to **B**, resulting in no phonon resonance. The opposite behavior in the Voigt geometry can also be understood because the incident light has an **E**_L_ component parallel to **B**, leading to a finite coupling between the electromagnetic wave and the induced phonon mode. Still, the oscillating **E**_L_ of unpolarized light has both component parallel to **B** and component perpendicular to **B**. To further verify our conclusion on the angle dependence, we study the reflectivity spectrum in the Voigt geometry in the presence of a linear polarizer while keeping the other experimental parameters the same. Figures 4c, d present the magneto-infrared spectra in the Voigt geometry with orthogonal polarizations. When the polarized **E**_L_ field is parallel to **B** (**E**_L_//**B**, Fig. 4c), the phonon resonance is clear and even stronger than the non-polarized case (Fig. 4b). On the other hand, the phonon resonance disappears in the cross-polarized situation (**E**_L_⊥**B**, Fig. 4d). Figure 4b of the unpolarized case is an average of the two polarized cases in Fig. 4c, d, therefore showing weaker amplitudes than those in Fig. 4c. The comparison here further confirms that the field-induced phonon activity is along the direction of **B**.

Next, we exclude the crystal anisotropy as a possible origin of the observed phenomena. First, the only difference between Fig. 4a, b is the magnetic field directions, thus the distinctive observations are unlikely attributed to the crystal anisotropy. Further, the NbAs crystal possesses the C_4v_ point group symmetry and we rotate the sample by 90° and keep other conditions identical to the case in Fig. 4c. The results are shown in Fig. 4e, where very similar phonon resonances are observed. If the absence of phonon activities in Fig. 4d came from the crystal anisotropy, these phonon activities would have also disappeared in Fig. 4e. Therefore, the crystal anisotropy is conclusively ruled out as the origin of the observed angular dependence of the phonon resonance. In Table 1, we summarize all the results from various geometries, which fully agree with the predictions based on the dynamic chiral anomaly^21,22^.

**Table 1:** Phonon activity in different experimental geometries.

Geometry	a	b	c	d	e
Faraday (F) or Voigt (V)	F	V	V	V	V
Angle between crystal [100] and B	⊥	||	||	||	⊥
Angle between crystal [100] and E	⊥	⊥||	||	⊥	⊥
Angle between B and E	⊥	⊥||	||	⊥	||
Phonon resonance	No	Weak	Yes	No	Yes

## Discussion

**Fig. 5:**
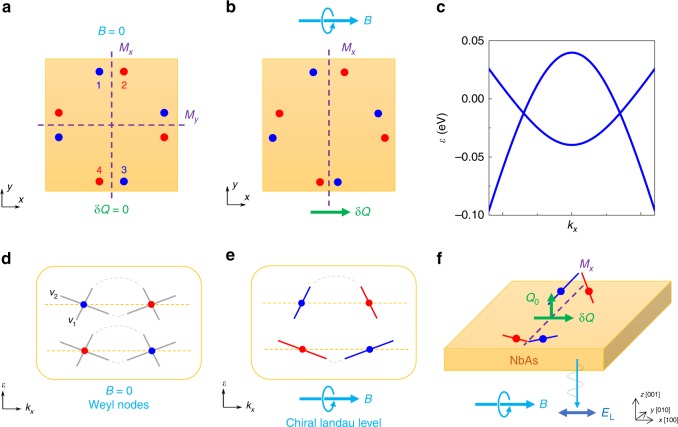
Crystal symmetry in the presence of magnetic fields. **a, b** Illustrations of the crystal symmetry. Without the **B** field, NbAs has *M*_*x*_ and *M*_*y*_ mirror planes. Weyl nodes are labeled with numbers. Chirality of Weyl nodes is denoted by the red and blue color. The presence of **B** field along the *x*-direction breaks *M*_*y*_, as illustrated by the shifting the Weyl nodes (not to scale in the plot). **c** Band structure calculated from a tight-binding model of NbAs class. The Fermi velocity of the two branches differs significantly in each Weyl node. **d** Weyl nodes without magnetic fields. **e** Formation of chiral Landau levels in the presence of **B** field, which leads to a chirality-dependent selection of the branch and difference in the Fermi velocities. **f** Experimental geometry and the magnetic-field-induced *M*_*y*_ symmetry breaking. In the presence of **B** field, different Weyl branches are chosen, allowing Weyl nodes 1 and 4 (2 and 3) to be different in their respective Fermi velocities. Therefore, the resultant phonon charge δ**Q** can be observed by parallel **E**_L_ (to **B**).

Having established a firm link between the phonon activity and the chiral anomaly in experiments, we now discuss how the dynamic chiral magnetic effect is allowed for the crystal symmetry of NbAs. In order to couple with the chiral magnetic current, the phonon charge **Q** of the phonon mode needs to transform as a pseudoscalar under the point group of the crystal, and changes sign under the mirror symmetries^21,22^. Therefore, no polarized phonon mode can have the magnetic-field-induced phonon activity in the presence of two or more non-parallel mirror planes, and NbAs was considered ineligible due to its two mirror planes along the [100] and [010] directions^21^. However, this symmetry argument was based on the zero-field crystal symmetries^26^. In fact, the presence of an external magnetic field will also break the improper rotation symmetries and lift the symmetry constraints, especially for the NbAs material family^20^. Let us consider the coupling of phonon charge δ**Q** that changes sign under x → −x, to the chiral magnetic current contributed by the Weyl nodes in NbAs with an external magnetic field along the *x* direction. Due to the persistent M_x_ symmetry, δ**Q** is a pseudoscalar. As shown in Fig. 5a, without the magnetic field, the NbAs crystal has two mirror planes denoted as *M*_*x*_ and *M*_*y*_ connecting the Weyl nodes in the *k*_*x*_–*k*_*y*_ plane labeled from 1 to 4. Similar arguments apply to the rest of the Weyl nodes elsewhere in the Brillouin zone of NbAs. The chirality of the Weyl nodes is denoted by the red and blue dots. Since both the pseudo-scalar phonon charge δ**Q** and the Weyl node chirality change sign under x → −x, the Weyl nodes 1 and 2 (3 and 4) connected by the *M*_*x*_ mirror symmetry contribute to the coupling additively. On the other hand, the contributions from Weyl nodes 1 and 4 (2 and 3) would cancel each other if *M*_*y*_ mirror symmetry was still present. However, the presence of the magnetic fields along the *x* direction breaks *M*_*y*_. Both the Zeeman effect and the Landau quantization lead to the location shifts of the Weyl nodes and thus, the differences in their Fermi velocities (Fig. 5b). A much more prominent influence comes from the distinctive difference between the Fermi velocities of the Weyl fermion’s two branches. Figure 5c presents the band structure calculated from the tight-binding model of the NbAs class. The two branches indeed have strongly anisotropic and largely distinctive Fermi velocities consistent with previous band structure studies^35,36^. The introduction of magnetic fields leads to the formation of the chiral Landau level as exhibited in Fig. 5e. The resulting in-field chiral channel descends from the original positive-x-moving or negative-x-moving Weyl fermions depending on its chirality and breaks the *M*_*y*_. It gives rise to the differences in the chiral dispersions and non-canceling contributions to the coupling between Weyl nodes (Fig. 5f). Therefore, a polarized pseudoscalar phonon mode can couple to the chiral anomaly in NbAs within the geometry of the experimental setup which stresses the importance of the magnetic-field-induced mirror symmetry breaking in Weyl semimetals. As we discuss in more detail in Supplementary Note 9 with group theory analysis, such a pseudoscalar phonon mode under the broken symmetry naturally descends from the E(3) phonons, which is nearly degenerate at **k** = 0 with the A_1_ phonon of the original crystal symmetries.

Besides Weyl semimetals, Dirac semimetals also possess chiral anomaly in the presence of magnetic fields. So the control of phonon charge by dynamic chiral anomaly is also expected in Dirac semimetals^21^. To confirm this possible universal behavior, we tested representative and well-studied Dirac semimetal Cd_3_As_2_ thin flakes. As shown in Supplementary Note 5 and Supplementary Fig. 2, although the phonon charge is non-zero at zero field, the magnetic-field-induced phonon charge is also observed in the A_1g_ mode^37^ of Cd_3_As_2_ with parallel **E**_**L**_ and **B**. In contrast, if **E**_**L**_ and **B** are perpendicular to each other, such a phonon charge contribution disappears in the Cd_3_As_2_ magneto-transmittance spectra. Therefore, the magneto-infrared study in Cd_3_As_2_ also agrees well with the theory of the phonon charge induced by the dynamic chiral anomaly and presents it as a general behavior in Dirac and Weyl semimetals. It is worth noting that the magnetic-field dependence of phonon intensity follows an identical **B**^4.3^ behavior which not only further proves the same origin of phonon behavior, but also indicates the strength of electron–phonon coupling being possibly tuned by magnetic fields (See Supplementary Fig. 5 and Supplementary Note 8 for details). On the other hand, in the real part of the optical conductivity spectrum, the extracted Lorentzian line shape and the monotonically increasing amplitude of the phonon modes suggest a negligible coupling between the magnetic-field-induced phonon and the inter-Landau-level tansitions. In principle, such coupling is not forbidden^38,39^ and can give rise to interesting phenomenon such as magneto-phonon resonance. We predict that in Weyl and Dirac semimetals with stronger electron–phonon coupling, the interplay between the field-induced phonon mode and the inter-Landau level transitions will lead to the strength oscillating or anti-crossing features of the induced phonon mode with respect to the magnetic fields.

In conclusion, the magneto-infrared spectroscopy is performed in Weyl semimetal NbAs. The AC *θ***E** · **B** is accessed by combining the internal phonon potential with the external magnetic field, leading to the chiral magnetic effect beyond the widely-investigated DC limit. In the presence of strong electron–phonon coupling, the original phonon being insensitive to IR spectroscopy can, therefore, be activated by magnetic fields. The anomalous phonon charge is parallel to the applied magnetic field, which can only be excited while the oscillating electric field has components parallel to the external magnetic field. Our results demonstrate an effective approach to access the dynamic chiral anomaly and allow for the further study of unique electromagnetic responses of Weyl semimetals in the AC regime.

## Methods

### Sample growth and optical characterization

The NbAs samples were grown by an enhanced method of chemical vapor transport^26^. Crystal structures were determined by X-ray diffraction. Raman spectra were measured at room temperature in a home-built optical system with an excitation wavelength of 633 nm. Infrared spectra were examined by in situ overcoating technique combined with Fourier transform infrared spectrometer.

### Magneto-infrared measurements

Magneto-infrared spectrum was measured in the far-infrared range with both Faraday and Voigt geometries in NHMFL, Tallahassee. The sample was kept at the liquid helium temperature and the magnetic field was applied. The light was shed on the sample near the incident angle. Therefore, one can still observe very subtle phonon features with orders of magnitudes lower amplitudes in the Faraday and perpendicular Voigt geometries. Reflectivity or transmittance signals were detected by a bolometer with spectra taken from Fourier transform infrared spectrometer.

## Supplementary information

Supplementary Information

## Data Availability

All data supporting the findings of this study are available from the corresponding author on request.
