# Supplementary material for: The discovery of dynamic chiral anomaly in a Weyl semimetal NbAs
Source: Nat Commun. 2020 Mar 6;11:1259. doi: 10.1038/s41467-020-14749-4 (PMC7060315; doi:10.1038/s41467-020-14749-4)
Supplement: Supplementary file 1 — Supplementary Information [file 41467_2020_14749_MOESM1_ESM.pdf]

Supplementary Information for

The discovery of dynamic chiral anomaly in a Weyl semimetal NbAs

Yuan et al.

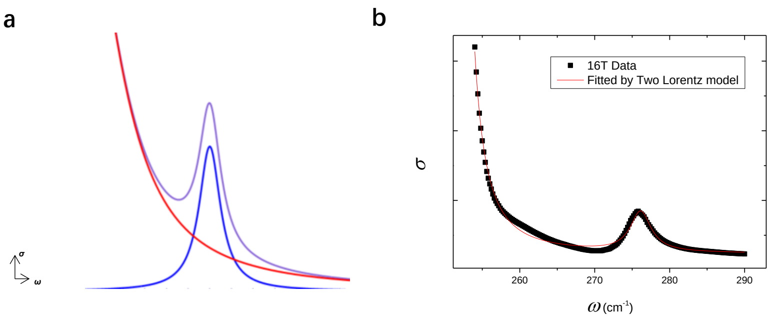

Supplementary Figure 1 | Line shape of the phonon mode. a, Schematic plot of two overlapping Lorentzian peaks. **b,** The fitting of the data at 16T with a parallel polarization geometry using two Lorentzian peak models.

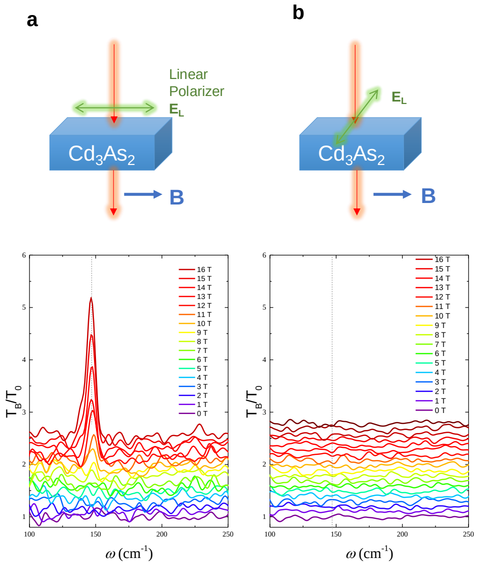

Supplementary Figure 2 | Magnetic-field-induced phonon charge in Dirac semimetal Cd_3As_2 as determined by the relative magneto-transmittance spectra T_B/T_0 in the Voigt geometries. **a, b,** The top panels exhibit the experimental geometries, and the bottom panels present the stacking plot of corresponding relative magneto-transmittance spectra. The phonon charge is observed only with parallel $\mathbf{E}_L$ and **B** components. The dashed line indicates the frequency of the induced phonon mode in parallel geometry. The phonon mode is IR-active at zero field. [1]

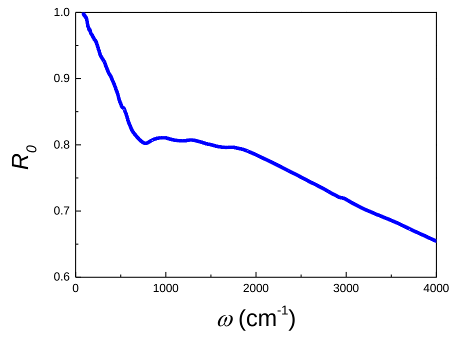

Supplementary Figure 3 | Optical spectra of NbAs (001) surface at zero field.

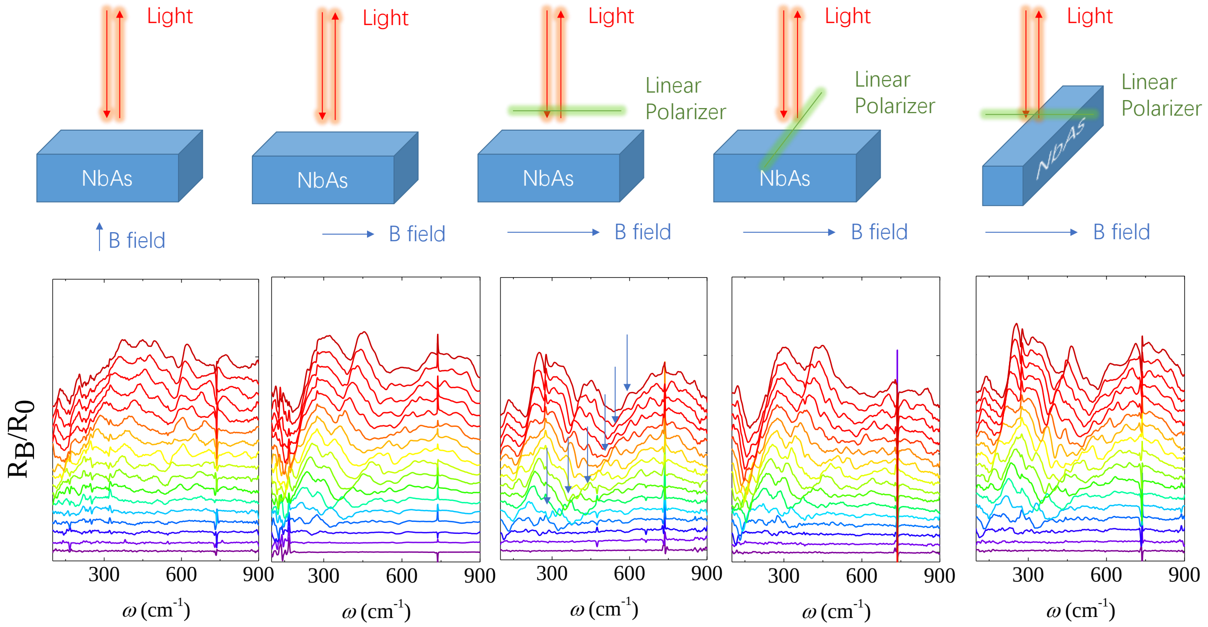

Supplementary Figure 4 | Magneto-optical spectra over a larger frequency range for NbAs at different geometries.

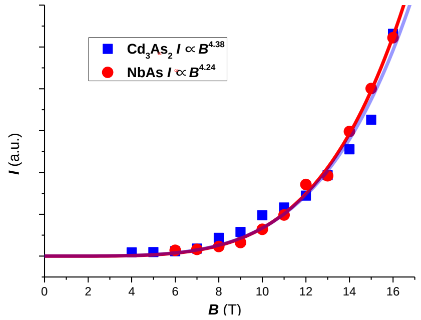

Supplementary Figure 5 | Phonon intensity versus magnetic field. Data points are extracted from the intensity of phonon peak and solid lines are the best fit to the power-law function.

Supplementary Note 1. The energy range of dynamic axial field

Experimental access to the oscillating axial field with the external field is challenging in Weyl semimetals due to two incompatible requirements. First, the photon energy cannot be higher than ~100 meV. Otherwise, it will exceed the energy range in which the effective Hamiltonian is able to describe Weyl fermions. Second, from supplementary figure 3, the electromagnetic field with energy lower than 100 meV will be screened due to the semi-metallic feature in bulky samples. Therefore, we turned to a more feasible alternative where the internal field helps to achieve the dynamic axial field.

Supplementary Note 2. The crystal orientation of NbAs

Based on the X-ray diffraction results in the main text, the lattice constants are calculated to be a=3.45Å and c=11.68Å, which are consistent with the previous studies.[2] A scanning electron microscope equipped with an energy-dispersive X-ray spectrometer was employed to detect the element composition, revealing a 1:1 ratio of Nb and As. For our NbAs crystal, the (101) crystal surface is smaller than the (001) surface, resulting in a larger noise in the infrared spectrum. Still, the relevant phonon mode is detected on the (101) surface. In comparison, the absence of phonon resonance even with the larger crystal surface and lower noise confirms the infrared inactivity of the A_1 mode in the (001) surface, consistent with our symmetry analysis. In the magneto-optical experiments, the (001) surface is chosen due to the inactivity of the A1 phonon at zero field.

Supplementary Note 3. The Kramers-Kronig analysis

The Kramers-Kronig analysis is performed to extract the conductivity spectrum. Here, the $R(B)/R(0)$ is measured under high fields with a frequency range from 80 to 6000 cm^{-1}. The zero-field spectrum $R(0)$ is measured by *in-situ* gold coating technique with a frequency range from 83 to 21500 cm^{-1}. In a large frequency range between 3000 and 6000 cm^{-1}, we found that $R(B)/R(0)$ is very close to 1 regardless of the magnetic field. Based on that, we assume that the magnetic field has a negligible influence on the

optical spectrum at high frequency. So, for the higher frequency, we can adopt $R(B) \approx R(0)$. Therefore, the effective frequency range for $R(B)$ in Kramers-Kronig analysis is 83 to 21500 cm^{-1}. For lower and higher frequency regimes, we use a typical extrapolation method as ref.[[3]] It is worth mentioning that the observed phonon activation behavior does not depend on the low/high-frequency extrapolation, which is a natural consequence of the original spectrum.

Supplementary Note 4. Lorentzian line shape in phonon resonance

The coexistence of the inter-Landau-level transitions and the field-induced phonon mode leads us to check the possible interplay between them. If the coupling exists between the inter-Landau-level transition and the phonon resonance, *i.e.*, a so-called magneto-phonon resonance, both the amplitude and the frequency of the phonon modes should shift periodically with the magnetic field. [4] This is in contrast to the spectrum in NbAs where the phonon intensity monotonically increases with the magnetic field and its frequency remains constant. Therefore, there is no signature of strong coupling between the phonon resonance and inter-Landau-level transitions. The optical conductivity spectrum reveals asymmetric features which are a natural consequence of two overlapping Lorentzian peaks. As shown in Supplementary Fig. 1a, the red and blue curves denote the Lorentzian peak from two uncoupled resonance. The red curve with a larger amplitude and width is from the inter-Landau-level resonance. The blue curve represents the magnetic-field-induced phonon resonance. While the symmetric Lorentzian peak is superimposed onto the tail of the red peak, the apparent conductivity peak (purple curve) appears to be asymmetric. In fact, the extracted data can be fitted using this model, as shown in Supplementary Fig. 1b. In summary, the observed phonon behavior is consistent with the picture of the dynamic chiral anomaly without coupling to the inter-Landau-level resonance.

Supplementary Note 5. Dynamic chiral anomaly in Dirac semimetal Cd_3As_2

Dirac semimetals can also host chiral anomaly due to the formation of non-degenerated Weyl nodes in the presence of magnetic fields. [5] The dynamic-chiral-anomaly-induced

phonon activity is predicted in Dirac semimetals such as Cd_3As_2. [6] So we study the magneto-optical properties of Cd_3As_2 thin plates. The experiment was performed on (112) surface of as-grown thin plates where the A_{1g} mode phonon is around 147 cm^{-1} and infrared-active at zero fields, [1] and we examine how the phonon mode evolves with the magnetic fields via optical transmittance spectroscopy. Supplementary Fig. 2 shows the relative transmittance spectra under different fields (0-16T) in different colors without stacking. With parallel $\mathbf{E}_L$ and **B**, the strength of the A1g phonon resonance is clearly modified by the **B** field (Supplementary Fig. 2a), which is consistent with the theory of the field-induced phonon charge. [6] In comparison, the phonon resonance can hardly be resolved with $\mathbf{E}_L$ perpendicular to **B** (Supplementary Fig. 2b). Both the field-dependent phonon strength and the angle-dependent behavior are consistent with the theoretical prediction. [6] Although the field-induced phonon charge is observed in both Cd_3As_2 and NbAs, the zero-field phonon charge is not zero in Cd_3As_2. As discussed in Supplementary Note IV, the field-induced phonon activity (as observed in NbAs) is much more unusual than the field-induced phonon intensity modulation (as observed in Cd_3As_2). Thus, the magneto-optic spectrum in NbAs serves as a more contrasting example of dynamic chiral anomaly control on lattice dynamics. However, the observation of phonon charge in Cd_3As_2 presents the field-induced phonon charge as a universal phenomenon in Dirac and Weyl semimetals.

Supplementary Note 6. Impact on phonon activity from the magnetic fields

Magnetic fields can easily influence the electronic states in condensed matters. However, it cannot readily and directly impact the phonon mode due to the large mass of the atoms and the protections from the lattice symmetries. In fact, the magnetic-field-induced phonon charge was observed in very few bulk systems, mostly in multiferroic materials. With the existence of ferromagnetic elements and the coupling to the ferroelectric state, the lattice change under magnetic fields results in the **B**-field dependent phonon. On the other hand, through extensive research in the NbAs family, there is no sign that multiferroic or even single-ferroic state exists in this crystal. Importantly, the magnetic-field-induced phonon charge mostly leads to the

modification of frequency or intensity an existing phonon mode. But to our best knowledge, the experimental observation of **B**-field-induced infrared phonon activity from an inactive phonon is highly unusual, suggesting a distinctive origin of phonon activity in NbAs.

Supplementary Note 7. Magneto-optical spectra at different geometries

The optical spectrum over a larger frequency range is shown in Supplementary Fig. 4, where the inter-Landau-level transitions can be well-resolved. For some of the resonance peaks as shown by the arrows, square-root-of-**B** dependence of the frequency is found which agrees with the picture of three-dimensional massless dispersions. The inter-Landau-level transitions may depend on the crystal anisotropy as well as the electric field polarization angle with respect to the magnetic field due to the optical selection rules at play. The latter dominates in our experiments because the signatures of the inter-Landau-level transition are almost identical for geometry c and e yet different for geometry d and e. The field-direction-dependent Landau levels and optical selection rules explain why the broad A-G peaks in the main text are different in these geometries.

Supplementary Note 8. Magnetic-field-dependent phonon intensity

In our spectrum, the intensity of A_1 mode increases with the magnetic field which qualitatively agrees with the chiral anomaly mechanism because the charge pumping rate is expected to increase with the magnetic field. In supplementary Fig. 4, we plot the normalized intensity of phonon mode from both NbAs (red) and Cd_3As_2 (blue). The intensity variations are very similar and both can be fitted well by the power law. Such a similar field-dependence indicates the same mechanism which is consistent with our conclusion. The intensity shows roughly $B^{4.3}$ dependence which exceeds the prediction of B^2 from the simple model [7]. Since the electron-phonon coupling can be tuned by magnetic fields, a possible mechanism is that electron-phonon coupling becomes stronger at higher fields.

Supplementary Note 9. Group theory analysis and the accidental degeneracy of the A_1 and E(3) phonons at k=0 in NbAs

At zero field, the crystal structure of NbAs is characterized by the space group $\boldsymbol{C_{4v}^{11}}$ with two Nb atoms and two As atoms per unit cell.

Supplementary Table 1: character table for point group $\boldsymbol{C_{4v}}$ and the unit cell atoms under symmetry transformations

$\boldsymbol{C_{4v}}$	E	$2C_4$	C_2	$2\sigma_v$	$2\sigma_d$
A_1	+1	+1	+1	+1	+1
A_2	+1	+1	+1	-1	-1
B_1	+1	-1	+1	+1	-1
B_2	+1	-1	+1	-1	+1
E	+2	0	-2	0	0
χ_{atom}	4	0	4	4	0

Looking up the character table for $\boldsymbol{C_{4v}}$, we have $\chi_{vector} = A_1 + E$. Also, the space group has a spatial translation following C_4 and σ_d, which maps the two Nb (As) atoms into each other. Therefore, we have $\chi_{atom} = 2(A_1 + B_1)$, and $\chi_{phonon} = \chi_{atom} \otimes \chi_{vector} = 2A_1 + 2B_1 + 4E$ at **k**=0, which contains 3 branches of acoustic modes $A_1 + E$ and 9 branches of optical modes $A_1 + 2B_1 + 3E$. Previous polarized Raman study for $\mathbf{e^i}$ and $\mathbf{e^s}$ in the xy plane has unambiguously pinpointed the A_1, B_1(2) and B_1(1) modes, at 268.2 cm^{-1}, 249.0 cm^{-1} and 231.7 cm^{-1}, respectively[8]. Ideally, the polarized Raman study in the zx configuration only responds to the E modes. However, four clear peaks are visible[1] at 149.2 cm^{-1}, 232.8 cm^{-1}, 249.7 cm^{-1} and 268 cm^{-1}. The former two can be unambiguously assigned to the E(1) and E(2) modes, respectively, leaving only the energy of E(3) to be determined. The additional peaks at 249.7 cm^{-1} and 268 cm^{-1} may be due to the slight misalignment of $\mathbf{e^i}$ and $\mathbf{e^s}$ away from perfect right angle, and the phonon modes A_1, B_1(2) with strong Ramon signals leave a mark in the otherwise-forbidden spectrum [8,9], and E(3) overlaps with one of them, leaving two ambiguous scenarios, see Supplementary Table 2. Note that this degeneracy is only at **k**=0 and is not guaranteed by systematic symmetries.

Supplementary Table 2: two ambiguous scenarios for the E(3) phonon energy as measured by the zx configuration of the polarized Raman study [8]

**Energy (k**=0)	249 cm^{-1}	268 cm^{-1}
Scenario 1	B_1(2) +E(3)	A_1
Scenario 2	B_1(2)	A_1+E(3)

Also, similar phenomena between A_1, B_1(2), and E(3) are observed on TaAs [8,9] with crystal structure identical to NbAs. Indeed, for all of the phonon modes A_1, B_1(2), and

E(3), the main atoms are the common As atoms between TaAs and NbAs. We note that the listed results in Ref. 1 have assumed the first scenario [8]. On the other hand, first-principle calculations for TaAs yielded very close energy between A_1 and E(3) at **k**=0, while the calculated energy of $B_1(2)$ is notably different [9], which favors the second scenario.

In the presence of a magnetic field B_x in the x-direction, only one of the reflection symmetries σ_v, namely M_x, survives. We note that the IR polarization E_x is odd under $\mathbf{M_x}$ transformation, therefore the selection rule requires the phonon representations to have pseudoscalar B' component to behave IR-active, irrespective of the detailed mechanism and dynamics of the phonons, *e.g.*, dynamic chiral anomaly, electron-phonon interactions, etc.

Supplementary Table 3: character table for $\mathbf{M_x}$ after the magnetic-field-induced symmetry breaking and the original phonon modes A_1 and $A_1 + E$.

$\mathbf{M_x}$	E		σ_v
A'	+1		+1
B'	+1		-1
A_1	+1		+1
$A_1 + E(3)$	+3		+1

Let's focus on the A_1 phonon energy where our main results in the main text occur. Interestingly, the lone A_1 phonon in the first scenario remains a scalar phonon A' during the symmetry breaking, therefore its IR activity is forbidden. On the other hand, the (nearly) degenerate $A_1 + E(3)$ phonon modes in the second scenario naturally split into 2A'+B', allowing responses to E_x photons. The experimental observation of phonon activities brings clear evidence to the second scenario. Therefore, despite the insignificant E(3) phonon charge and the dominant A1 phonon's **Q** along z at zero field, a **B**-induced $\delta\mathbf{Q}$ is allowed to have an observable x-component even in the presence of a residue M_x mirror plane.

Supplementary References

1 Akrap, A., Hakl, M., Tchoumakov, S., Crassee, I., Kuba, J., Goerbig, M. O., Homes, C. C., Caha, O., Novak, J., Teppe, F., Desrat, W., Koohpayeh, S., Wu, L., Armitage, N. P., Nateprov, A., Arushanov, E., Gibson, Q. D., Cava, R. J., van der Marel, D., Piot, B. A., Faugeras, C., Martinez, G., Potemski, M. & Orlita, M. Magneto-Optical Signature of Massless Kane Electrons in Cd_3As_2. *Phys Rev Lett* **117**, 136401 (2016).

2 Boller, H. & Parthé, E. The transposition structure of NbAs and of similar monophosphides and arsenides of niobium and tantalum. *Acta Crystallographica* **16**, 1095–1101 (1963).

3 Xu, B., Dai, Y. M., Zhao, L. X., Wang, K., Yang, R., Zhang, W., Liu, J. Y., Xiao, H., Chen, G. F., Trugman, S. A., Zhu, J. X., Taylor, A. J., Yarotski, D. A., Prasankumar, R. P. & Qiu, X. G.

Temperature-tunable Fano resonance induced by strong coupling between Weyl fermions and phonons in TaAs. *Nature communications* **8**, 14933 (2017).

4 Goler, S., Yan, J., Pellegrini, V. & Pinczuk, A. Raman spectroscopy of magneto-phonon resonances in graphene and graphite. *Solid State Communications* **152**, 1289-1293 (2012).

5 Armitage, N., Mele, E. & Vishwanath, A. Weyl and Dirac semimetals in three-dimensional solids. *Reviews of Modern Physics* **90**, 015001 (2018).

6 Song, Z., Zhao, J., Fang, Z. & Dai, X. Detecting the chiral magnetic effect by lattice dynamics in Weyl semimetals. *Physical Review B* **94** (2016).

7 Hui, A., Zhang, Y. & Kim, E.-A. Optical signatures of the chiral anomaly in mirror-symmetric Weyl semimetals. *Physical Review B* **100**, 085144 (2019).

8 Liu, H. W., Richard, P., Zhao, L. X., Chen, G. F. & Ding, H. Comparative Raman study of Weyl semimetals TaAs, NbAs, TaP and NbP. *Journal of physics. Condensed matter : an Institute of Physics journal* **28**, 295401 (2016).

9 Liu, H. W., Richard, P., Song, Z. D., Zhao, L. X., Fang, Z., Chen, G. F. & Ding, H. Raman study of lattice dynamics in the Weyl semimetal TaAs. *Physical Review B* **92** (2015).
